# Effects of a novel DNA methyltransferase inhibitor Zebularine on human lens epithelial cells

**Published:** 2012-01-10

**Authors:** Peng Zhou, Yi Lu, Xing-Huai Sun

**Affiliations:** Department of Ophthalmology, Eye and ENT Hospital of Fudan University, Shanghai, China

## Abstract

**Purpose:**

Posterior capsular opacification (PCO) is a common long-term complication of modern cataract surgery. We have shown that Zebularine, an inhibitor of DNA methylation, suppresses transforming growth factor-β (TGFβ)-induced lens epithelial cells (LECs)–myofibroblasts transdifferentiation. The purpose of this study is to evaluate the role that Zebularine plays in the inhibition of PCO pathogenesis, including its effect on attachment, migration, and proliferation of LECs in vitro.

**Methods:**

A tetrazolium dye–reduction assay (MTT test) was performed to determine cell proliferation. Cell attachment was assessed by modified MTT test. Migration was determined by the transwell method after incubation of LECs with Zebularine. The effect of Zebularine on DNA-methyltransferase 1 (DNMT1), phospho-p44/42 Map Kinase, and protein kinase B (Akt) were analyzed by western blot.

**Results:**

Zebularine was an effective inhibitor of human LEC proliferation, attachment, and migration in vitro. A Zebularine concentration of 100 μM accounted for the following: inhibition of cell proliferation of 57.2%, reduction in cell attachment to 29.6%, and inhibition of cell migration of 58.9%. All effects were dose dependent. Zebularine treatment resulted in dose-dependent decreases of DNMT1, phosphorylated p44/42 MAP Kinase, and phosphorylated Akt.

**Conclusions:**

Zebularine is capable of inhibiting the crucial cellular events in PCO pathogenesis in vitro. Zebularine acts through the inhibition of DNMT1, and it consequently down regulation of the expression of proliferative and survival genes that relate to pathogenesis of PCO. These findings suggest that Zebularine may become a therapeutic approach for the prevention of PCO.

## Introduction

The continuous improvements in surgical technique, intraocular lens (IOL) design, and IOL material have significantly reduced the incidence of posterior capsule opacification (PCO) in the past 30 years [1]. However, PCO remains the most common long-term complication of modern cataract surgery [2,3]. Decreased visual acuity induced by PCO is reported to occur in 20% to 40% of patients 2 to 5 years after surgery [3,4]. As a routine treatment for PCO, neodymium: YAG (Nd:YAG) laser capsulotomy is performed successfully in many patients. However, there are complications such as retinal detachment, damage to the intraocular lens, and cystoid macular edema [5,6].

Cataract surgery induces a wound-healing response in the lens, and residual lens epithelial cells (LECs) undergo an epithelial-to-mesenchymal transition (EMT), followed by enhanced proliferation, migration, and collagen deposition. Therefore, postsurgical medical inhibition of LECs EMT, proliferation, and migration is a possible option for preventing PCO.

Epigenetic modifications are post-transcriptional, reversible events that do not target gene sequence, and inhibition of these mechanisms could theoretically be advantageous in the treatment of fibrosis disease [7,8]. As a consequence, the role of epigenetic regulators like histone deacetylase (HDAC) and DNA methyltransferase (DNMT) inhibitors as a treatment for PCO is under evaluation.

Zebularine is a cytidine analog containing a 2-(1H)-pyrimidinone ring that was originally developed as a cytidine deaminase inhibitor. It acts primarily as a trap for DNMT protein by forming tight covalent complexes between DNMT protein and Zebularine-substituted DNA [9]. In contrast to other DNMT inhibitors, it is quite stable [10,11] and low toxicity [12-14]. Preclinical studies using Zebularine have shown favorable toxicity and stability profiles, making it an attractive candidate for epigenetic treatment of PCO [13].

The purpose of this study was to determine whether Zebularine is capable of inhibiting the crucial cellular events for PCO formation (i.e., human LEC proliferation, attachment, and migration) in vitro.

## Methods

The institutional review board (IRB) of Fudan University Eye and ENT Hospital, Shanghai, China approved our use of cultured human LECs. All procedures conformed to the Declaration of Helsinki for research involving human subjects. The Zebularine we used was a kind gift from Dr. Victor E. Marquez (Laboratory of Medicinal Chemistry, National Cancer Institute, Frederick, MD).

### Cell culture

HLE B-3 cells, an immortalized cell line derived from infant human lens tissue and transformed with adenovirus 12-simian virus (SV40), were obtained from ATCC (Rockville, MD) and cultured in Eagle’s minimum essential medium (GIBCO BRL, Grand Island, NY) with 20% fetal bovine serum, 100 units/ml penicillin, and 100 mg/ml streptomycin at 37 °C in a humidified 5% CO_2_ atmosphere.

### Cell proliferation assay

HLE B-3 cells were seeded in 96-well plates (150 μl/well at a density of 5×10^3^ cells/well in DMEM containing 10% FBS). After 24 h, Zebularine (10, 50, and 100 μM) was added. After another 12, 24, 48, 72, and 96 h, cells were washed gently twice with PBS, and fresh medium (150 μl) was added to each well with MTT (3-(4, 5-dimethyl-2-thiazolyl)-2, 5-diphenyl-2H tetrazolium bromide, 5mg/ml, 20 µl; Sigma, St. Louis, MO). After 4 h of incubation, the supernatants were decanted, and the formazan precipitates were solubilized by the addition of 150 µl of 100% DMSO (Sigma) and placed on a plate shaker for 10 min. Absorbance at 550 nm was determined on a multi-well plate reader (Benchmark plus™; Bio-Rad, Tokyo, Japan). The number of proliferated cells was proportional to the absorbance of MTT at 550 nm. All experiments were performed at least three times.

### Cell attachment assay

The attachment assay was performed by a modified MTT assay using 96-well plates coated with fibronectin. After treatment with Zebularine (10, 50, and 100 μM) for 48 h, LECs were trypsinized and re-suspended in DMEM with 0.4% FBS. One hundred microliters of cell suspension (10^4^ cells) were added to each well and allowed to attach for 5 and 15 min. The cells were washed gently twice with PBS, and fresh medium (150 µl) was added to each well with MTT (5 mg/ml, 20 µl; Sigma). After 4 h of incubation, the supernatants were decanted, and the formazan precipitates were solubilized by the addition of 150 µl of 100% DMSO (Sigma) and placed on a plate shaker for 10 min. Absorbance at 550 nm was determined on a multi-well plate reader (Benchmark plus™; Bio-Rad). The number of attached cells was proportional to the absorbance of MTT at 550 nm. All experiments were performed at least three times.

### Cell migration assay

Migration was measured using Transwell (Costar, Cambridge, MA) assay, as previously described [15]. Briefly, 5×10^4^ LECs were seeded in the upper part of a chamber in 24-well plates after treatment with Zebularine (10, 50, and 100 μM) for 48 h, with inserts coated with fibronectin (2 µg/cm^2^). The lower chamber was filled with 0.4% FBS-DMEM containing 20nng/ml recombinant PDGF (R&D Systems Inc., Minneapolis, MN). After 5 h incubation, the inserts were washed three times with PBS, fixed with cold methanol (4 °C) for 10 min, and counterstained with hematoxylin for 20 min. The number of migrated cells was counted by phase-contrast microscopy (200×). Three randomly chosen fields were counted per insert. All experiments were performed at least three times.

### Western blot assay

Confluent cells grown in 6-well plates in DMEM with 0.4% FBS were lysed, supernatants were collected, and proteins were resolved on Tris-HCl 10% polyacrylamide gels at 120 V. The proteins were transferred to PVDF blotting membrane (Millipore, Bedford, MA). The membranes were probed with antibodies for DNA-methyltransferase 1 (DNMT1; Abcam, Cambridge, MA), phospho-p44/42 MAP Kinase (Thr 202/Thy 204; Cell Signaling, Danvers, MA), phospho-AKT (Ser 473; Cell Signaling), and glyceraldehyde 3-phosphate dehydrogenase (GAPDH) (Abcam), respectively, all at 1:1,000 dilution. Membranes were washed and incubated with a horseradish peroxidase (HRP)-conjugated secondary antibody (1:3,000; Vector Laboratories, Burlingame, CA) for 30 min at room temperature. Images were developed using ECL chemiluminescence detection solution (Amersham Pharmacia Biotech, Cleveland, OH). All experiments were performed at least three times.

### Statistics

The data were analyzed using Student’s *t*-test, and a p<0.05 was accepted as significant.

## Results

### Zebularine inhibited LECs proliferation in a dose- and time-dependent manner

**Figure 1:**
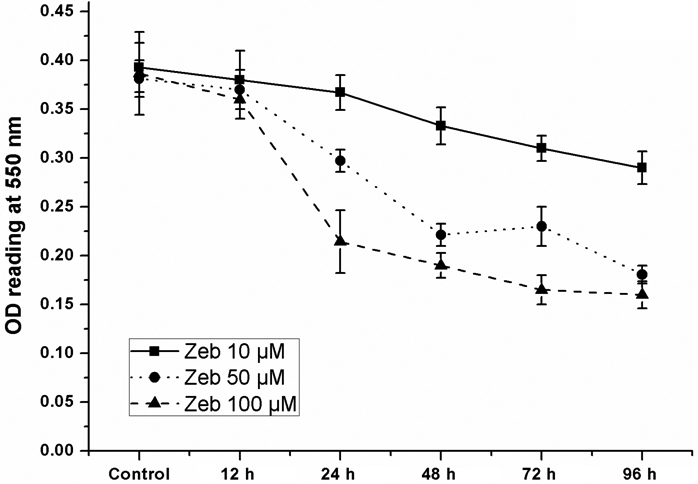
Zebularine inhibited LECs proliferation in a dose- and time-dependent manner. The human LECs were treated with various concentrations of Zebularine and the percentage of surviving cells was determined by MTT assay from 12 to 96 h. A time- and dose-dependent decrease in proliferation was seen with the application of Zebularine (10, 50, and 100 μM; p<0.01). A Zebularine concentration of 100 μM accounted for the inhibition of cell proliferation of 57.2% in 96 h.

The human LECs were treated with various concentrations of Zebularine, and the percentage of surviving cells was determined by MTT assay from 12 to 96 h. A time- and dose-dependent decrease in proliferation was seen with the application of Zebularine (10, 50, and 100 μM; p<0.01). As shown in Figure 1, 25.6±4.1% inhibition of cells occurred with 10 μM Zebularine at 96 h, 52.6±2.3% inhibition with 50 μM Zebularine, and 57.2±3.3% inhibition with 100 μM Zebularine.

### Zebularine decreased LECs attachment

**Figure 2:**
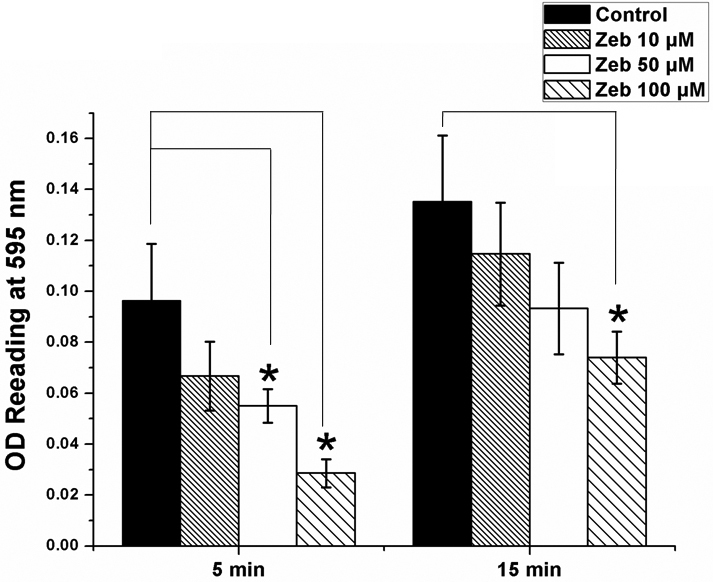
Zebularine decreased LECs attachment in a dose-dependent manner. LECs attachment was measured using modified MTT test. A dose-dependent decrease in attachment was seen with the application of high concentrations of Zebularine (50 μM and 100 μM). LECs attachment was significantly reduced to 29.6% in the early phase (5 min) of cell attachment. *p<0.01, Error bar indicates mean±SEM.

LECs attachment was measured using modified MTT test (Figure 2). After 48 h exposure to Zebularine, no statically significant difference was found in LECs attachment between control and low-dose Zebularine (10 μM)-treated cells. However, a dose-dependent decrease in attachment was seen with the application of higher concentrations of Zebularine (50 μM and 100 μM; p<0.01). LECs attachment was significantly reduced by 70.4±5.3% in the early phase (5 min) of cell attachment (p<0.01).

### Zebularine inhibited LECs migration

**Figure 3:**
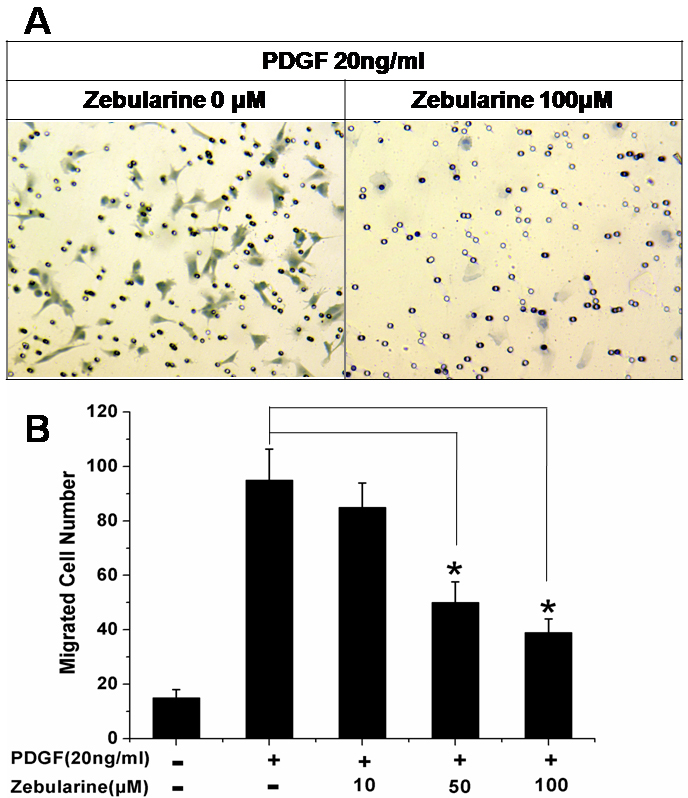
Zebularine inhibited LECs migration in a dose-dependent manner. LECs migration to the lower compartment of a transwell was significantly increased in response to PDGF stimulation. PDGF-induced LECs migration was inhibited by Zebularine (**A**) in a dose-dependent manner (**B**). The maximal inhibition (58.9±6.1%) was seen at 100 μM Zebularine (p<0.01). *p<0.01, Error bar indicates mean±SEM.

LECs migration to the lower compartment of a transwell was significantly increased in response to PDGF stimulation (Figure 3). PDGF-induced LECs migration was inhibited by Zebularine (50 and 100 μM) in a dose-dependent manner. The maximal inhibition (58.9±6.1%) was seen at 100 μM Zebularine (p<0.01).

### Zebularine inhibited the epigenetic regulator

**Figure 4:**
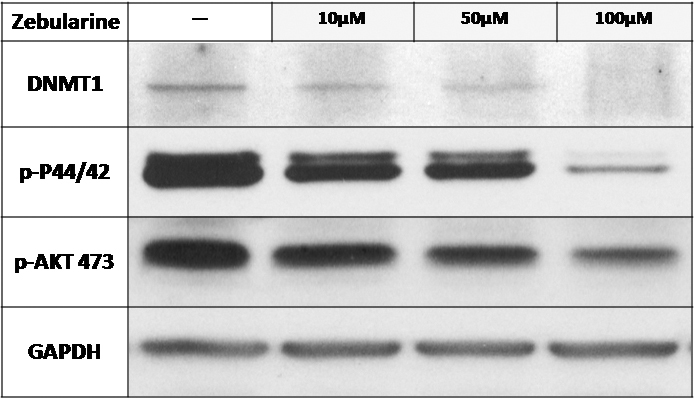
The effect of Zebularine on the expression of methylation related genes and genes involved in the pathogenesis of PCO. Zebularine treatment was associated with a dose-dependent depletion of DNMT1 in all three doses (10, 50, and 100 μM). Zebularine inhibited the phosphorylation of Akt at serine 473. Phosphorylated p44/42 MAPK level decreased by 87.5% after treatment with high dose (100 μM) Zebularine in 48 h.

Because of Zebularine’s activity as a DNMT inhibitor in other model systems, its effect on expression of DNMT1 in LECs was examined. As expected, Zebularine treatment was associated with a dose-dependent depletion of DNMT1 in all three doses (10, 50, and 100 μM; Figure 4).

### Zebularine inhibited the cellular survival genes

Akt is involved in cellular survival pathways and is active after being phosphorylated at serine 473. In this study, Zebularine inhibited Akt phosphorylation at serine 473 in all three doses. Mitogen-activated protein kinases (MAPKs) are a widely conserved family of serine/threonine protein kinases involved in many cellular programs such as cell proliferation, differentiation, motility, and death. The p44/42 MAPK signaling pathway can be activated in response to a diverse range of extracellular stimuli including mitogens, growth factors, and cytokines. In this study, phosphorylated p44/42 MAPK level decreased by 87.5% after treatment with high dose (100 μM) Zebularine for 48 h (Figure 4).

## Discussion

In this study, we found that Zebularine is capable of inhibiting the crucial cellular events in PCO pathogenesis in vitro. After a single exposure to Zebularine added to the cell-culture medium in the presence of serum, HLE-B3 proliferation, attachment, and migration were effectively inhibited.

The initial cellular events in PCO can be described as the migration and attachment of LECs to the posterior capsule surfaces and their following proliferation. In this study, Zebularine effectively inhibited LECs attachment and migration. Inhibition of the initial stages in PCO development may prevent the disease from progressing to the more advanced stages commonly associated with a severe reduction in visual acuity.

The effect of Zebularine on cell migration was reported by Hellebrekers et al. [16]. However, they reported that migration of endothelial cells was not significantly influenced by treatment with Zebularine at concentrations up to 500 μM. This difference may be due to different type of cells and methods used in different experiments. Hellebrekers et al. [16] used human umbilical vein endothelial cells (HUVEC) and scratched wound assay in response to Zebularine treatment, while we used fetal LECs and transwell assay. Hellebrekers’s method more likely showed a wound healing response, while the transwell assay method showed the effect of Zebularine on LECs chemotaxis response. In addition, the pretreatment with Zebularine was 48 h in our experiment, while Hellebrekers et al. [16] pretreated the cells for 72 h.

Proliferation is another important event in the pathogenesis of PCO. Our results demonstrate that Zebularine effectively inhibited LECs proliferation in a time- and dose-dependent manner. Previous studies showed that Zebularine induces apoptotic cell death in many cell lines. Scott et al. [13] analyzed the effects of Zebularine on cell proliferation in acute myeloid leukemia cells (AML193 cell line) using MTT and tritiatedthymidine assays. They found that treatment of AML193 cells with Zebularine concentrations necessary to demethylate DNA causes a decrease in cell viability and proliferation, and an increase in the percentage of apoptotic cells and cells arrested in G_2_/M of the cell cycle.

Calvisi et al. [17] reported that treatment with the demethylating agent Zebularine caused marked activation of MST1, p38MAPK, and JNK in hepatoma cell lines. Neureiter et al. [14] found that higher concentrations of Zebularine led to rapid induction of apoptosis in YAP C, DAN G, and Panc-89 pancreatic adenocarcinoma cells; lower concentrations remained ineffective in these cell lines. Billam et al. [18] found that Zebularine inhibits human breast cancer cell growth in a dose- and time-dependent manner.

Pharmacologic PCO prevention represents a valuable option, especially for refractive and pediatric cataract surgery. The current therapeutic concepts for pharmacologic PCO prevention are compromised in terms of potential clinical application. The effective agents, represented by mitomycin-C [19] and 5-fluorouracil [20], require a sealed-capsule irrigation device due to significant toxic side effects [21]. Methylation inhibitor is a new strategy for treating disease. Methylation inhibitor is well tolerated and safe, according to a recent phase I clinical studies [22-24]. Further phase II clinical trials are undergoing. Other DNA methylation inhibitors, such as 5-Aza-C and 5-Aza-dC, have shown significant clinical activity against tumors. However, the clinical use of these agents is complicated by their toxicity and instability in aqueous solutions [13]. The half-life of Zebularine is much longer, and its toxicity is much lower. Therefore, Zebularine offers a better option for potential clinical application.

**Figure 5:**
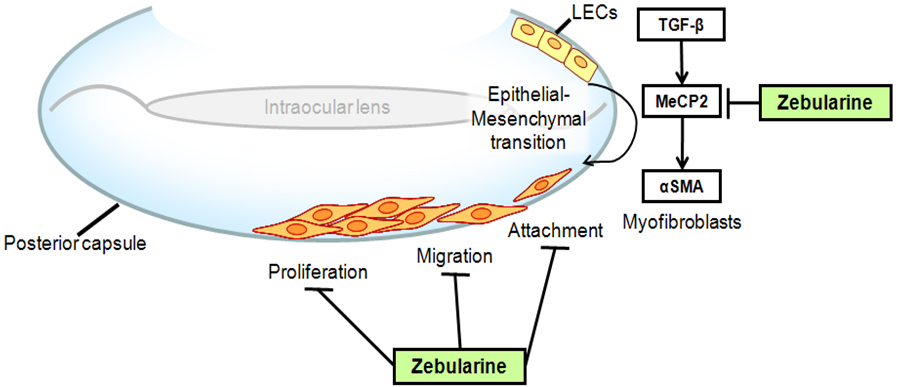
Model for Zebularine inhibiting PCO. We have shown that Zebularine inhibited MeCP2, and furthermore, inhibits TGFβ-induced α-SMA expression. In this study, we found that Zebularine is capable of inhibiting the crucial cellular events in PCO pathogenesis, including LECs proliferation, attachment, and migration. Therefore, Zebularine could potentially prevent PCO formation. LECs: lens epithelial cells; IOL: intra ocular lens.

We have shown that Zebularine suppressed TGFβ2-induced lens epithelial cell–myofibroblast transdifferentiation [7]. In this study, our data demonstrate that Zebularine is an effective demethylating agent. Zebularine can inhibit LECs proliferation, attachment, and migration in vitro. These findings suggest that Zebularine may become a new option for preventing PCO after conventional cataract surgery (Figure 5).
